# Adolescent polycystic ovary syndrome according to the international evidence-based guideline

**DOI:** 10.1186/s12916-020-01516-x

**Published:** 2020-03-24

**Authors:** Alexia S. Peña, Selma F. Witchel, Kathleen M. Hoeger, Sharon E. Oberfield, Maria G. Vogiatzi, Marie Misso, Rhonda Garad, Preeti Dabadghao, Helena Teede

**Affiliations:** 1 Discipline of Paediatrics, The University of Adelaide Robinson Research Institute and Endocrine Department, Women’s and Children’s Hospital, 72 King William Road, North Adelaide, SA 5006 Australia; 2 Department of Pediatrics, Division of Pediatric Endocrinology, UPMC Children’s Hospital of Pittsburgh, University of Pittsburgh, Pittsburgh, PA USA; 3 Department of OBGYN, University of Rochester Medical Center, Rochester, NY USA; 4 Division of Pediatric Endocrinology, Columbia University Irving Medical Center, New York, NY USA; 5 Division of Endocrinology, Children’s Hospital of Philadelphia, Perelman School of Medicine at the University of Philadelphia, Philadelphia, PA USA; 6 Monash Centre for Health Research and Implementation, School of Public Health and Preventive Medicine, Monash University and Monash Health, Melbourne, VIC Australia; 7 Department of Endocrinology, Sanjay Gandhi Postgraduate Institute of Medical Sciences, Lucknow, India

**Keywords:** Adolescents, Girls, Polycystic ovary syndrome, Evidence-based, Diagnosis, Treatment

## Abstract

**Background:**

Diagnosing polycystic ovary syndrome (PCOS) during adolescence is challenging because features of normal pubertal development overlap with adult diagnostic criteria. The international evidence-based PCOS Guideline aimed to promote accurate and timely diagnosis, to optimise consistent care, and to improve health outcomes for adolescents and women with PCOS.

**Methods:**

International healthcare professionals, evidence synthesis teams and consumers informed the priorities, reviewed published data and synthesised the recommendations for the Guideline. The Grading of Recommendations, Assessment, Development, and Evaluation (GRADE) framework was applied to appraise the evidence quality and the feasibility, acceptability, cost, implementation and strength of the recommendations.

**Results:**

This paper focuses on the specific adolescent PCOS Guideline recommendations. Specific criteria to improve diagnostic accuracy and avoid over diagnosis include: (1) irregular menstrual cycles defined according to years post-menarche; > 90 days for any one cycle (> 1 year post-menarche), cycles< 21 or > 45 days (> 1 to < 3 years post-menarche); cycles < 21 or > 35 days (> 3 years post-menarche) and primary amenorrhea by age 15 or > 3 years post-thelarche. Irregular menstrual cycles (< 1 year post-menarche) represent normal pubertal transition. (2) Hyperandrogenism defined as hirsutism, severe acne and/or biochemical hyperandrogenaemia confirmed using validated high-quality assays. (3) Pelvic ultrasound not recommended for diagnosis of PCOS within 8 years post menarche. (4) Anti-Müllerian hormone levels not recommended for PCOS diagnosis; and (5) exclusion of other disorders that mimic PCOS. For adolescents who have features of PCOS but do not meet diagnostic criteria an ‘at risk’ label can be considered with appropriate symptomatic treatment and regular re-evaluations. Menstrual cycle re-evaluation can occur over 3 years post menarche and where only menstrual irregularity or hyperandrogenism are present initially, evaluation with ultrasound can occur after 8 years post menarche. Screening for anxiety and depression is required and assessment of eating disorders warrants consideration. Available data endorse the benefits of healthy lifestyle interventions to prevent excess weight gain and should be recommended. For symptom management, the combined oral contraceptive pill and/or metformin may be beneficial.

**Conclusions:**

Extensive international engagement accompanied by rigorous processes honed both diagnostic criteria and treatment recommendations for PCOS during adolescence.

## Background

Polycystic ovary syndrome (PCOS) is the most common endocrine condition affecting between 8 and 13% of women of reproductive age [1] and 6–18% of adolescent girls [2, 3] depending on the diagnostic criteria used and the population studied [4–6]. Adolescence, as defined by the World Health Organisation, is the period between 10 and 19 years of age that includes significant and critical changes in growth, development and puberty.

Diagnosis of PCOS during adolescence is both controversial and challenging due to the overlap of normal pubertal physiological changes (irregular menstrual cycles, acne and polycystic ovarian morphology on pelvic ultrasound) with adult PCOS diagnostic criteria. These challenges have been acknowledged in adult and paediatric consensus statements [4–10]. Specifically, challenges include the risk of under-diagnosis, delayed and/or poor diagnosis experiences [11], and over-diagnosis as well as the additional risk of the use of inconsistent non-evidence-based approaches in the diagnosis and management of PCOS among specialists, general practitioners and allied health professionals [12–14]. These challenges are exacerbated by the lack of robust evidence. For example, ‘only adolescents’ studies are limited and suboptimal in quality. Further, adult studies that include adolescents do not specify the number of adolescents or time post menarche, which is critical information to determine the evolution of normal pubertal physiological changes [15]. Lastly, relevant consensus statements are often not specific to adolescents and/or not based on robust high-level evidence and/or rigorous processes, indicating the need for high quality, evidence-based guidelines [4, 5, 7, 10].

The aim of the first International Evidence-based Guideline for the Assessment and Management of PCOS (‘the Guideline’) was to promote the accurate diagnosis of PCOS, optimal consistent care, the prevention of complications and improved patient health outcomes from adolescence to adulthood [6]. The term ‘adolescence’ in the Guideline was defined as the period between 10 and 19 years of age according to the World Health Organisation. However, based on evidence, those who were within a gynaecological age of 8 years or less than 8 years post menarche were also identified in the recommendations.

This paper focuses specifically on the adolescent recommendations from the Guideline and examines the evidence and rationale supporting these recommendations. We discuss the importance of avoiding missed diagnosis, of delayed, under- or over-diagnosis, and of evidence-based management to address PCOS symptoms in adolescents. Additionally, we expand on approaches to identify girls ‘at risk’ of PCOS but not yet diagnosed, including the need for future follow-up.

## Methods

Best practice evidence-based guideline development methodology was applied [16–18], as detailed in the full Guideline and Technical Report that are freely available [19, 20]. The Guideline development process engaged health professional societies and consumer organisations, multidisciplinary experts including paediatricians and gynaecologists who are experts in adolescent gynaecology, and women with PCOS, who were directly involved at all stages.

As previously outlined, a six-continent international advisory and project board, a multidisciplinary international guideline development group, and consumer and translation committees provided governance of the Guideline processes [6]. The engaged, international society-nominated panel provided experts in endocrinology, paediatrics, gynaecology, reproductive endocrinology, obstetrics, and public health, alongside consumers and project management, evidence synthesis, and translation experts. A total of 37 societies and organisations representing 71 countries were engaged in a 15-month process. The Guideline addressed 60 prioritised clinical questions encompassing 40 evidence-based reviews and 20 narrative reviews, most of which sought to identify evidence relevant to adolescents. The detailed description of the methods of development of each of the 60 questions and subsequent reviews is extensive and cannot be provided herein; therefore, for each topic outlined, we have provided guidance to the corresponding section of the Guideline Technical Report [20]. Appraisal of Guidelines for Research and Evaluation (AGREE) II-compliant processes were followed, with extensive evidence synthesis. The Grading of Recommendations, Assessment, Development, and Evaluation (GRADE) framework was applied across evidence quality, desirable and undesirable consequences, feasibility, acceptability, cost, implementation and, ultimately, recommendation strength [16]. Evidence-based recommendations were formulated on the basis of an extensive and detailed review of all available evidence and following a rigorous, structured GRADE processes as well as extensive international peer review.

Importantly, extensive engagement with stakeholders, including women with PCOS, and the multidisciplinary panels informed the Guideline research questions and highlighted the impact of PCOS across the lifespan. It also highlighted the importance of accurate and early diagnosis to optimise education, treatment and the prevention of longer-term complications.

Categories of recommendations are described in Table 1 and included evidence-based recommendations, consensus recommendations and practice points [6]. Key recommendation terms include ‘should’, ‘could’ and ‘should not’. These terms are informed by the nature of the recommendation (evidence or consensus), the GRADE framework and evidence quality, and are independent descriptors reflecting the judgement of the multidisciplinary Guideline Development Group (GDG), including consumers (women with PCOS). These terms refer to overall interpretation and practical application of the recommendation, balancing benefits and harms. ‘Should’ is used where benefits of the recommendation exceed harms and where the recommendation can be trusted to guide practice. ‘Could’ is used where either the quality of evidence was limited or the available studies demonstrate little clear advantage of one approach over another, or where the balance of benefits to harm was unclear. ‘Should not’ is used where there is either a lack of appropriate evidence or the harms may outweigh the benefits.

**Table 1:** Categories of recommendations in the Polycystic Ovary Syndrome (PCOS) Guideline

Evidence-based recommendations	Evidence-based recommendations are made where evidence is sufficient to inform a recommendation made by the guideline development group
Consensus recommendations	Clinical consensus recommendations are made in the absence of adequate evidence on PCOS; these are informed by evidence in other populations and is made by the guideline development group, using rigorous and transparent processes
Practice points	Clinical practice points are made where evidence was not sought and where important clinical issues arose from discussion of evidence-based or clinical consensus recommendations

The GRADE quality of the recommendation was determined by the GDG from structured consideration of the GRADE framework [16], including desirable effects, undesirable effects, balance of effects, resource requirements and cost effectiveness, equity, acceptability, and feasibility. The GRADE approach included Conditional recommendation against the option; Conditional recommendation for either the option or the comparison; Conditional recommendation for the option; and Strong recommendation for the option.

The quality of the evidence was categorised according to information about the number and design of studies addressing the outcome; judgments about the quality of the studies and/or synthesised evidence such as risk of bias, inconsistency, indirectness, imprecision and any other considerations that may influence the quality of evidence; key statistical data; and classification of the importance of the outcomes (Table 2). The quality of evidence reflects the extent to which our confidence in an estimate of the effect is adequate to support a particular recommendation [18] and was largely determined by the expert evidence synthesis team.

**Table 2:** Categories of quality (certainty) of evidence (adapted from GRADE [16])

High	Very confident that the true effect lies close to that of the estimate of the effect
Moderate	Moderate confidence in the effect estimate – the true effect is likely to be close to the estimate of the effect, but there is a possibility that it is substantially different
Low	Limited confidence in the effect estimate – the true effect may be substantially different from the estimate of the effect
Very low	Very little confidence in the effect estimate – the true effect is likely to be substantially different from the estimate of effect

The GRADE recommendations affirm that the quality of evidence is a continuum; any discrete categorisation involves a degree of arbitrariness [16]. Nevertheless, the advantages of simplicity, transparency and vividness outweigh these limitations [18]. Consensus recommendations do not have a quality of evidence rating (as no evidence was found) and practice points do not have a ‘GRADE’ rating as they arose from discussion of evidence-based or clinical consensus recommendations (Table 1). The meaning or interpretation of the GRADE quality of recommendations is provided in Table 3 [16, 21].

**Table 3:** Strength of recommendations (adapted from GRADE and ESHRE Manual)

Target group	Strong recommendations^a^	Conditional (weak) recommendations for the option (test or treatment)	Conditional (weak) recommendation for either the option or the comparison	Research-only recommendations	Clinical practice points^b^
Consumers	Most people in your situation would want the recommended course of action and only a small proportion would not	The majority of people in your situation would want the recommended course of action but some would not	There is considerable lack of clarity over whether the majority of people in your situation would want the recommended course of action or not	The test or intervention should only be considered by patients and clinicians within the setting of a research trial for which appropriate approvals and safety precautions have been established	Clinicians, patients and policy-makers are informed on the clinical implications relevant to implementation of recommendations
Health professionals	Most patients should receive the recommended course of action	Recognise that different choices will be appropriate for different patients and that greater effort is needed with individuals to arrive at management decisions consistent with values and preferences Decision aids and shared decision-making are important here		The test or intervention should only be considered by patients and clinicians within the setting of a research trial for which appropriate approvals and safety precautions have been established	
Policy-makers	The recommendation can be adopted as policy in most situations	Policy-making needs to consider perspectives and involvement of diverse stakeholders	Policy decisions remain unclear	Policy-makers need to be aware of the need for evidence gaps and health professional and consumer-prioritised research gaps	

^a^ Strong recommendations based on high quality evidence will apply to most patients for whom these recommendations are made, but they may not apply to all patients in all conditions; no recommendation can take into account all of the often-compelling unique features of individual patients and clinical circumstances

^b^ A clinical practice point is developed by the Guideline Development Group to support recommendations; advice can be provided to enhance shared decision-making and on factors to be considered in implementing a specific test or intervention

Following completion of these stages, the draft guideline was distributed to all 37 partnering societies, including paediatric endocrine societies, and to specifically formulated special interest groups in each society providing review and feedback. Evidence was required to support any requests for recommendation modifications. The feedback and responses are available under the Guideline supporting documents (Public Consultation Comments and Developer Responses) [19]. These were reviewed by a specially convened paediatric guideline committee (including the authors of this paper). This process further refined the recommendations, which in turn were approved by all societies.

## Results

The full Guideline is summarised by Teede et al. [6] and provides recommendations on the five areas covered by the GDGs, namely diagnosis and comorbidities risk assessment, assessment and treatment of emotional wellbeing, lifestyle, pharmacological treatment for non-fertility indications, and assessment and treatment of infertility.

This paper examines the recommendations related to adolescents, including diagnosis, assessment and treatment of emotional wellbeing, and lifestyle and pharmacological treatment for non-fertility indications. We do not cover assessment and treatment of infertility, which can be found in the Guideline. The recommendations provided here include evidence-based recommendations, consensus recommendations and practice points, with most of the evidence-based recommendations in adolescents being related to the treatment of PCOS.

### Diagnosis

Key changes within the Guideline recommendations include the elimination of specific unnecessary tests and the importance of identifying adolescents ‘at risk’ of PCOS. The changes also aim to avoid misdiagnosis or delayed, under- or over-diagnosis. Adolescent consensus recommendations for PCOS diagnosis are based on the best-quality available evidence and its limitations.

#### Criteria required

1. *Irregular menstrual cycles and ovulatory dysfunction*

Clear definition of irregular menstrual cycles according to time post menarche was the first strong consensus recommendation as there was limited evidence to formulate an evidence-based recommendation (Table 4). When irregular menstrual cycles are present, a diagnosis of PCOS should be considered.

**Table 4:** Definition of irregular menstrual cycles in adolescents according to time post menarche

Time post menarche	Definition of irregular menstrual cycles
Less than 1 year post menarche	Irregular menstrual cycles are normal pubertal transition
> 1 to < 3 years post menarche	< 21 or > 45 days
> 3 years post menarche	< 21 or > 35 days or < 8 cycles per year
More than 1 year post menarche	> 90 days for any one cycle
	Primary amenorrhoea by age 15 years or > 3 years post thelarche (breast development)

Whilst the literature search on the question “At what time point after the onset of menarche do irregular menstrual cycles indicate ongoing menstrual dysfunction?” revealed 36 studies (see Section 1.1 in the Technical Report), these did not meet the selection criteria for the systematic review framework as they did not report on the duration of menstrual irregularity and/or diagnosis of PCOS. Hence, relevant information from diverse studies was extracted from the literature in a narrative review format. This informed the consensus recommendation based on the evidence on menstrual cycles in healthy girls [22–32].

This recommendation was also informed by previous available guidelines, multidisciplinary expertise, consumer perspectives, international feedback, and the potential for both over and delayed diagnosis when assessing this diagnostic feature in PCOS. This recommendation considered available data on normal physiological events during adolescence such as menstrual cycle interval variations according to time post menarche and the fact that anovulation is a common physiological event in early post menarcheal years [22–34]. Physiological maturation of the hypothalamic–pituitary ovarian axis occurs over the years and ovulation and menstrual cycles in adolescents may not match those of women in reproductive age [26, 28, 33, 34]. More specific cut-offs aligned with gynaecological maturity were provided in comparison to previous guidelines [4, 5, 9].

Another practice point from the Guideline is that ovarian dysfunction can still occur in adolescents or women with regular menstrual cycles and, if anovulation is suspected, serum progesterone levels can be measured to confirm it.

2. *Hyperandrogenism*
  - *Biochemical*

Calculated free testosterone, free androgen index or bioavailable testosterone should be used to assess biochemical hyperandrogenism in the diagnosis of PCOS. This was the only strong evidence-based recommendation of low GRADE quality for biochemical testing for hyperandrogenism, including one study in adolescents [35]. This recommendation was determined after examination of the literature showing that androgen levels in adolescents reach adult levels around the time of menarche. Seven studies evaluated the most effective measure to diagnose PCOS-related hyperandrogenism (biochemical) [35–41], with only one of these studies performed in adolescents, including 89 subjects (26 with girls with PCOS and 63 controls) [35] (see Section 1.2 in the Technical Report).

There were two conditional evidence-based recommendations of low GRADE quality derived from studies in adult women that reported on the diagnostic accuracy of different hormone markers to detect PCOS [36–41]. One study compared the diagnostic accuracy of different types of assay to detect PCOS but did not include adolescents [42]. High-quality assays, such as liquid chromatography–mass spectrometry and extraction/chromatography immunoassays, should be used for the most accurate assessment of total or free testosterone in PCOS. Androstenedione and dehydroepiandrosterone sulfate (DHEAS) provide limited additional information in the diagnosis of PCOS, however they could be considered if total or free testosterone are not elevated. Androstenedione and DHEAS are more useful in excluding other causes of hyperandrogenism. Androstenedione is elevated in non-classical adrenal hyperplasia. DHEAS is a predominantly adrenal androgen and mild elevations can be seen in PCOS, whereas significant elevations and/or virilisation can be seen in androgen-secreting adrenal tumours.

Important practice points included avoiding the assessment of biochemical hyperandrogenism in women on hormonal contraception (a drug withdrawal of 3 months is recommended, addressing needs for contraception through other means). Careful interpretation of androgen levels is needed by considering the reference ranges of the laboratory used and normal values derived from a well-phenotyped population, including age and pubertal-specific stages. Additionally, the guidelines noted that, when clinical hyperandrogenism is not detected, biochemical hyperandrogenism should be determined using appropriate high-quality assays.

b. *Clinical hyperandrogenism*

A comprehensive history and physical examination should be completed for symptoms and signs of clinical hyperandrogenism, which in adolescents include severe acne and hirsutism; this was the first strong consensus recommendation because no evidence-based recommendation in relation to clinical hyperandrogenism was available. This recommendation was based on the fact that mild comedonal acne is common in adolescent girls but moderate or severe comedonal acne (i.e. 10 or more facial lesions) in early puberty or moderate to severe inflammatory acne during the peri-menarcheal years is uncommon (less than 5%) and is more likely to relate to clinical hyperandrogenism [43–45]. In addition, there are no studies in adolescents evaluating alopecia in the context of PCOS.

Standardised visual scales are preferred when assessing hirsutism, such as the modified Ferriman–Gallwey score (mFG), with a level ≥ 4–6 indicating hirsutism, depending on ethnicity, and acknowledging that self-treatment is common and can limit clinical assessment. This second strong consensus recommendation was based on the most common visual assessment tool, the mFG [46, 47]. This tool was first used in a cohort of women that included 75 females aged between 15 and 24 years, yet the number of adolescents per se was not specified [46]. mFG evaluates terminal hairs (hairs that would grow more than 5 mm in length if left intact and are usually pigmented and medullated) in 9 primarily androgen-dependent areas – upper lip, chin and neck, upper chest (excluding the nipples), upper abdomen (above the umbilicus), lower abdomen, thighs (front and/or back), upper back, lower back, and upper arms. Each area is visually scored from zero (no terminal hair visible) to four (terminal hair consistent with a well-developed male) [46, 47]. Inquiry regarding self-treatment should be made and recorded in the examination. The definition of ‘abnormal’ in hirsutism is controversial and varies across ethnicities. The original Ferriman–Gallwey cut-off was 4–6 and this later evolved in the literature to an arbitrary cut-off of 6–8 based on the 95th percentile of unselected women (which likely included women with PCOS) [48]. Lower mFG cut-offs are generated in studies where the lower 85th to 90th percentile cut-offs are used to define ‘normal’ or from cluster analysis including other features of PCOS (> 3 in White and Black women [49], > 5 in Mongoloid Asian (Han Chinese) women [50]). Over 50% of women with mFG scores of 3–5 and over 70–90% of women with mFG scores > 5 have elevated androgens and/or PCOS. Of note, these cut-offs sought accurate diagnosis by avoiding under-diagnosis and reflected evidence on clustering of hyperandrogenism with other PCOS features, rather than arbitrary percentile cut-offs. However, the cut-offs are based mainly on studies in adult women, not adolescents (see Section 1.3 in the Technical Report). Of note, three well-designed population studies, including a total of 889 healthy adolescents and some adolescents with PCOS (12.5–17.5 years), have demonstrated that higher hirsutism scores are related to higher testosterone levels [2, 51, 52]. Additionally, a longitudinal study in adolescents showed an increase in hirsutism scores with age and time post menarche [53].

Health professionals should be aware of the potential negative psychosocial impact of clinical hyperandrogenism. Reported unwanted excess hair growth should be considered important, regardless of apparent clinical severity. This third consensus recommendation was based on a systematic review of quality of life and included a study with 97 adolescents with PCOS [54].

#### Investigations not recommended

1. *Pelvic ultrasound for PCOS diagnosis*

Pelvic ultrasound should not be used for the diagnosis of PCOS in those with a gynaecological age of < 8 years (< 8 years post menarche) due to the high incidence of multi-follicular ovaries in this life stage. This was a strong consensus recommendation and not an evidence-based recommendation because reliable data regarding longitudinal ovarian morphology are limited. Additionally, the specific 8 years post-menarche cut-off was based on a consensus recommendation by the GDG, consulted with international societies and refined after international peer review. The recommendation also considered 15 studies evaluating the most effective ultrasound criteria to diagnose PCOS in women using various measures and thresholds for ovarian volume and follicle number. However, adult PCOS criteria have been derived from transvaginal ultrasounds, which should be avoided in females not yet sexually active. Three of these 15 studies included adolescents (181 with PCOS and 137 controls), two of which used trans-abdominal ultrasound and one used trans-rectal ultrasound (68 with PCOS and 26 controls) [35, 55, 56]. The 12 studies included women aged between 18 and 40 years (average minimum and maximum ages 24.9 and 31.2 years, respectively) (see Section 1.4 in the Technical Report). Additionally, this recommendation considered the physiological maturation of the hypothalamic–pituitary ovarian axis [26, 28, 33, 34].

Furthermore, a polycystic ovarian morphology may represent a marker of PCOS or may be normal in young women. A population study showed a higher prevalence of polycystic ovarian morphology in very young women without menstrual irregularity and hyperandrogenism (although this study used older criteria for polycystic ovarian morphology and none of the criteria for polycystic ovarian morphology have used age base cut-offs) [57]. A small longitudinal study of healthy adolescents at 2 to 4 years post menarche suggested that polycystic ovarian morphology is common and not associated with reproductive dysfunction [58]. Due to this overlap between follicle numbers per ovary in normal adolescents and in those with other features of PCOS, the adult polycystic ovarian morphology criteria are likely inaccurate for the ultrasound diagnosis of PCOS during adolescence. Additionally, the use of pelvic ultrasound will increase the risk of PCOS over-diagnosis during adolescence. Lastly, the Guideline also highlighted the significant evolution of ultrasound technologies and the evolving number of follicles recommended for diagnosis even in adult women, a factor that contributed to the recommendation to avoid the use of pelvic ultrasound in the diagnosis of PCOS in females at less than 8 years post menarche. The term ‘adolescents’ was avoided in this recommendation and the terms ‘gynaecological age’ or ‘time post menarche’ were used to align with international paediatric guidelines describing menstrual irregularity [4, 32]. A gynaecological age of < 8 years as the cut-off was in part chosen based on normative models suggesting that the maximum ovarian volume is reached at age 20 [59]. There are no large studies across the lifespan to validate normal ovarian development with normal reference ranges. Some studies indicate that ovarian volume changes over time with increased antral follicles and stroma; ovarian size increases from ages 9 to 11, reaching the maximum volume at age 20 [59–62]. Although pelvic ultrasound is not indicated for PCOS diagnosis in adolescents, it can be used to investigate other possible uterine or ovarian abnormalities in adolescent girls such as those that present with primary amenorrhoea.

2. *Anti-Müllerian hormone (AMH)*

AMH is a polypeptide of the transforming growth factor-β family secreted by granulosa cells of the pre-antral and small antral ovarian follicles. Hence, AMH has been evaluated in the diagnosis of PCOS, especially in situations where pelvic ultrasound is not feasible [63, 64].

AMH levels should not be used as an alternative for the detection of polycystic ovarian morphology or as a single test for the diagnosis of PCOS. This evidence-based recommendation (conditional and of low GRADE quality) was based on 29 studies, including a systematic review [65] evaluating whether AMH was effective for the diagnosis of PCOS or polycystic ovarian morphology. Six of the 29 studies [58, 66–70] were performed in adolescents from different countries and included 375 adolescent girls diagnosed with PCOS and 639 controls. One study was in overweight/obese adolescents [70] and one study did not specify body mass index (BMI) [69]. Only one study in adolescents evaluated the diagnostic accuracy of AMH for polycystic ovarian morphology [58] (see Section 1.5 in the Technical Report).

Although serum AMH levels in adolescents and adult women with both polycystic ovarian morphology and PCOS are significantly higher than in those without these features in all the studies included [58, 65–70], there was significant overlap in levels as well as heterogeneity between studies with regards to assays, life stage and phenotypes of the populations studied, including the use of different PCOS criteria.

There is emerging evidence that, with improved standardisation of assays and established cut-off levels based on large populations of different ages and ethnicities, AMH assays will become more accurate in the detection of polycystic ovary morphology.

#### Exclusion of other conditions

The diagnosis of PCOS is a diagnosis of exclusion – all other aetiologies that can cause menstrual irregularities and/or hyperandrogenism must be excluded, regardless of the fact that some aetiologies are less common in adolescents. The most important cause of amenorrhoea in a sexually active adolescent is pregnancy. Menstrual irregularity alone could be due to gonadotropin deficiency caused by functional hypothalamic amenorrhoea, secondary deficiency due to any systemic cause, or to a primary gonadotropin defect. Many conditions can lead to hyperandrogenism; the most common albeit rare condition (outside of PCOS) is non-classic congenital adrenal hyperplasia (NCAH), characterised by a marked elevation of androgen levels. Deficiency of 21-hydroxylase, due to mutations in the 21-hydroxylase (*CYP21A2*) gene, is the most common type of NCAH seen. The prevalence of NCAH is reported as 1 in 1000 but is even more frequent in certain ethnic groups [71]. Diagnosis of NCAH is suspected if an adolescent girl has clitoromegaly and/or an early-morning, follicular phase 17-hydroxyprogesterone (17-OHP) level of > 200 ng/dl (> 6 nmol/L) and confirmed at 17-OHP levels of > 35 nmol/L (1000–1500 ng/dl) 60 min after administration of 250 μg of synthetic adrenocorticotropic hormone or synacthen [72].

Hypothyroidism, hyperprolactinemia, glucocorticoid excess due to Cushing’s disease, glucocorticoid resistance, and androgen-secreting ovarian or adrenal tumours can cause menstrual irregularity and/or hyperandrogenism [73]. A thorough history and physical examination to look for signs of hypothyroidism, galactorrhoea, glucocorticoid excess or virilisation are important in the evaluation of an adolescent girl with suspected PCOS. Measurement of serum thyroid stimulating hormone, prolactin, gonadotropins, androgen and/or follicular phase 17-OHP levels is required to exclude these conditions. If the androgen levels are twice above the upper limit of the reference range, imaging is also required to assess the ovary and/or adrenals. An overnight dexamethasone suppression test or midnight salivary cortisol estimation is required to exclude Cushing’s syndrome, though importantly only where the condition is clinically suspected.

#### Adolescents ‘at risk’ of PCOS

A practice point for this section is that, for adolescents who have features of PCOS but do not meet the diagnostic criteria, the ‘at risk’ of PCOS label could be considered and reassessment is advised at or before full reproductive maturity. This timing is at 3 years post menarche in relation to menstrual cycle irregularity and at 8 years post menarche in relation to the use of pelvic ultrasound to identify a polycystic ovarian morphology, as described in the ultrasound section. The timing of reassessment in relation to menstrual cycles was based on physiological maturation of the hypothalamic–pituitary ovarian axis and the likelihood of having ovulatory cycles matching those of women in reproductive age [26, 28, 33, 34]. Reassessment is particularly important for adolescent girls with persisting PCOS features and those with significant weight gain in adolescence and should occur after hormonal therapy washout of at least 3 months if this therapy has been commenced (whilst ensuring contraceptive needs are met). This recommendation is based on the need to address isolated symptoms, such as irregular menstrual cycles or clinical hyperandrogenism, where the diagnosis of PCOS remains unclear as pelvic ultrasound is not recommended at this life stage. PCOS cannot be diagnosed during adolescence unless both irregular menstrual cycles or hyperandrogenism are present. It was recommended that these adolescents be made aware of their ‘at risk’ status for PCOS and that future re-evaluation may be needed if both features persist beyond 3 years post menarche. This recommendation emerged from published evidence as well as from strong and consistent feedback from women with PCOS, who had a poor diagnostic experience and often failed to be diagnosed during adolescence, remaining unaware of this potential diagnosis until seeking treatment for infertility [11] (see Section 1.1 in the Technical Report). Adult physicians and reproductive endocrinologists also expressed significant concern over delayed diagnosis. All GDGs highlighted the need to acknowledge ‘risk’ yet avoid over-diagnosis or premature labelling. Another practice point for this section is that, in adolescents with irregular menstrual cycles and ‘at risk’, the value and optimal timing of assessment and diagnosis of PCOS should be discussed with the adolescent and their families, considering all diagnostic challenges at this life stage as well as psychosocial and cultural factors.

### Emotional wellbeing

The first consensus recommendation was that ‘Health professionals should be aware that, in PCOS, there is a high prevalence of moderate to severe anxiety and depressive symptoms in adults and a likely increased prevalence in adolescence’; the second consensus recommendation was that ‘Anxiety and depressive symptoms should be routinely screened in all adolescents and women with PCOS at diagnosis’. If the screening results are positive, further evaluation and/or referral for assessment and treatment should be completed by suitably qualified health professionals informed by regional guidelines. There were no evidence-based recommendations in the Guideline for the prevalence, screening, diagnostic assessment or treatment of emotional wellbeing for women or adolescents with PCOS and the GDG provided the two strong consensus recommendations mentioned above with limited available evidence, most of which related to studies in women and only one study in adolescents [74, 75]. These recommendations were based on a growing body of literature that includes cross-sectional, longitudinal and population-based studies indicating that women with PCOS experience a high prevalence of anxiety and depression [75–82] and the fact that psychological issues are under-recognised [11]. According to previous and recent meta-analyses, the odds of moderate/severe depressive symptoms were increased as were the odds of moderate/severe anxiety symptoms, independent of obesity [7, 77, 83]. Similar evidence has started emerging in adolescent girls with PCOS, though the data are still rather limited [74, 75]. Emotional wellbeing during adolescence was examined in a meta-analysis and subgroup analysis observing that adolescent girls with PCOS scored higher in depressive and anxiety symptoms compared to controls; the standardised mean differences for depression and anxiety were 0.54 (95% CI 0.16–0.93) and 0.48 (95% CI, 0.00–0.96), respectively [75]. The clinical significance of these findings as well as the factors associated with scores for anxiety/depression are uncertain. In adult women with PCOS, increased BMI, insulin resistance, hyperandrogenaemia, worse hirsutism scores and infertility have been implicated in the development or exacerbation of depressive and anxiety symptoms [79]. Similar studies/data are lacking for adolescents (see Section 2.2 in the Technical Report).

Given the high rates of anxiety/depression in women with PCOS and the current evidence in adolescence, the Guideline recommends routine screening of these conditions in all adolescents and women at diagnosis, with further evaluation if the screen is positive. Rescreening is recommended according to medical judgement and the patient’s progress. Often, the physical features of PCOS can cause anxiety and depressive symptoms, yet over-diagnosis of these psychological conditions should be avoided whilst recognising the distress caused by PCOS and paying attention to factors that most influence quality of life. If clinical anxiety or depression are diagnosed, the individual should be treated according to standard guidelines as specific studies that evaluate the effect of psychiatric medications in PCOS are lacking (see Sections 2.8 and 2.9 in the Technical Report). The use of psychiatric agents may worsen obesity and should be used with caution. According to recent but limited data, cognitive behavioural therapy may be beneficial in terms of weight loss and symptoms of anxiety/depression in both adolescents and women with PCOS; however, the data merit further confirmation [83]. Similarly, initial outcome data regarding lifestyle changes on body weight and wellbeing in women with PCOS appear promising, while studies in adolescents are yet to be performed. Recent studies in women with PCOS indicate an increased prevalence of disordered eating and the international Guideline provides conditional recommendations for awareness, assessment and appropriate therapy of disordered eating [84] (see Section 2.5 in the Technical Report). Whether the same applies to adolescent girls with PCOS is yet to be determined.

### Treatment

#### Lifestyle

Lifestyle interventions (preferably multi-component, including diet, less sedentary behaviour, exercise and behavioural strategies) should be recommended in all those with PCOS and excess weight to achieve reductions in weight, central adiposity and insulin resistance. This was a conditional and low GRADE quality evidence-based recommendation for lifestyle interventions. Weight gain is the number one priority expressed by young women with PCOS [85]. Additionally, this recommendation was also based on the bidirectional association between PCOS and weight gain, wherein there is a high prevalence and important adverse impact of excess weight in PCOS [86]. Obesity may induce PCOS by worsening features and, although the concept of ‘secondary PCOS’ has been raised, it is still far from defined. The same argument could apply to PCOS secondary to other drivers of hyperinsulinemia such as type 1 diabetes. We could elude to an emerging concept of PCOS secondary to other drivers of hyperinsulinaemia and hyperandrogenism such as obesity and type 1 diabetes [4, 87]. Vitally important was the emphasis on lifestyle interventions to prevent excess weight gain in adolescents with PCOS, avoiding waiting until obesity has developed, where lifestyle intervention alone is unlikely to reverse the excess adiposity. Specific recommendations on regular weighing, whilst acknowledging appropriate sensitivities, were made. Once present, obesity exacerbates metabolic and psychological comorbidities in adolescents with PCOS [88, 89]. Additionally, weight gain escalates from adolescence and early adulthood requiring early vigilance and intervention [90, 91]. Two systematic reviews of lifestyle interventions in women with PCOS show improvements in weight, hyperandrogenism and insulin resistance [92, 93]. In particular, in adolescents with PCOS, a multidisciplinary model of care with a dietitian, health psychologist and endocrinologist showed that a behavioural intervention enhanced weight loss when combined with dietary intervention compared to receiving neither or dietary advice only [94]. Additionally, a randomised controlled trial (RCT) comparing lifestyle intervention (diet, physical activity and behaviour) with placebo over 24 weeks had some benefits in adolescents with PCOS [95] (see Chapter 3 in the Technical Report).

#### Pharmacological principles of treatment in PCOS

Recommendations for the pharmacological treatment of adolescents with PCOS include the use of the combined oral contraceptive pill (COCP) and/or metformin in those with a clear diagnosis or in adolescents deemed ‘at risk’ of PCOS for the management of symptoms. These recommendations were made following review of the evidence for the efficacy of COCP, metformin and antiandrogens alone or in combination for the management of hormonal or clinical PCOS features in adolescents and adult women with PCOS. These recommendations were also based on the need for interventions and longitudinal follow-up to address PCOS symptoms in adolescents who are ‘at risk’ but do not yet meet full diagnostic criteria. Whilst diagnosis is recommended where both irregular menstrual cycles and hyperandrogenism are present, when only one of these is detected, symptom reduction and future reassessment was deemed preferable to prematurely labelling or over-diagnosing an adolescent as having PCOS. This approach addressed the conundrum that healthcare professionals involved in the medical management of adolescent girls face in potentially over-diagnosing normal pubertal girls, girls with transient menstrual irregularities, or under-diagnosing girls who will likely evolve to a PCOS phenotype. Additionally, medications such as COCP are commonly prescribed in adolescents for contraception and other indications.

The following practice points should apply to all medications used in PCOS to inform adolescents and their families and to guide health professionals when considering or recommending pharmacotherapy: (1) an individual’s personal characteristics, preferences and values are of importance when recommending pharmacotherapy; (2) the benefits, adverse effects and contraindications in PCOS and in general populations should be considered; and (3) the fact that COCPs, metformin and other pharmacological treatments are generally ‘off-label’ for the treatment of PCOS should be discussed where allowed, with adolescents and their families to consider the evidence and potential side effects for each option. However, off-label use is predominantly evidence based for other conditions and is allowed in many countries. Off-label prescribing occurs when a medication is prescribed for an indication, a route of administration, or a patient group that is not included in the approval product information document for that medication by the relevant regulatory body. Prescribing off-label is unavoidable and most commonly means that there has not been a submission to request evaluation of the indication or of that patient group for any given medication. The latter is very common in paediatric practice. Off-label use of metformin in adolescents with PCOS has been described [96].

Holistic approaches are required and pharmacotherapy in PCOS should be considered alongside education and counselling, lifestyle, and other options, including cosmetic therapy. Of note, the Guideline did not review the evidence on cosmetic therapies for hirsutism or non-hormonal therapies for acne.

#### Medications

1. *COCP (oestrogen and progestin preparations)*

The COCP alone should be considered in adolescents with a clear PCOS diagnosis or could be considered in those deemed ‘at risk’ but not yet diagnosed with PCOS in both groups for the management of clinical hyperandrogenism and/or irregular menstrual cycles. These two conditional and low GRADE quality evidence-based recommendations were based on limited evidence in adolescents, including two RCTs [95, 97] and a meta-analysis [98]. The latter evaluated the effect of metformin versus the COCP and included four RCTs [95, 99–101] with a total of 170 adolescents aged 12–21 years, showing improvements in menstrual irregularities and acne. The RCTs were of 6-months duration with the exception of one, which was of 24 months [101] (see Section 4.2 and 4.3 in the Technical Report). Evidence on COCP use from the general population also informed the Guideline by including considerations of adverse effects. Although the COCP is relatively safe, there are absolute medical contraindications to consider according to World Health Organisation Guidelines such as history of migraine with aura, deep vein thrombosis, pulmonary embolism, known thrombogenic mutations, multiple risk factors for cardiovascular disease, breast cancer, neuropathy, severe cirrhosis and malignant liver tumours [102]. Smoking and obesity are also risk factors for deep vein thrombosis; however, the absolute risk of these complications in adolescents remains very low.

Specific types or doses of progestins, oestrogens or combinations of COCP cannot currently be recommended due to insufficient data among women and adolescents with PCOS. Practice should be informed by general population guidelines. This was a conditional and low GRADE quality evidence-based recommendation in relation to the use of specific types of COCP and was based on evidence of COCP use in the general population and World Health Organisation Guidelines as there were no trials evaluating this in women or adolescents with PCOS (see Section 4.2 and 4.3 in the Technical Report). A practice point for this section includes that COCPs with 35 μg of ethinylestradiol and cyproterone acetate should not be used as first-line therapy due to the absence of evidence of greater efficacy and the presence of higher risks, including deep venous thrombosis. All COCPs are associated with an increased risk of deep venous thrombosis, but the risk is higher with COCPs containing 30–35 μg of ethinylestradiol and gestodene, desogestrel, cyproterone acetate or drospirenone when compared to the COCP containing 30 μg of ethinylestradiol with levonorgestrel, norethisterone (norethindrone) or norgestimate. Lower-risk COCP preparations should be recommended as first-line therapy [103].

2. *Combined COCP and metformin*

The COCP in combination with metformin could be considered in adolescents with PCOS and a BMI > 25 kg/m^2^ where the COCP and lifestyle changes do not achieve desired goals. This was a conditional and low GRADE quality evidence-based recommendation based on a single RCT including 79 adolescents followed over 6 months [95] and supported by 6 RCTs in adult women that highlighted the differential effects according to BMI categories [104]. Although the combination of metformin and the COCP offers additional benefits, these did not surpass the impact of the COCP plus lifestyle interventions and hence the combination was indicated when the COCP and lifestyle interventions have failed to meet the treatment goals. Since the COCP in combination with metformin lead to mild gastrointestinal side effects, these potential side effects need to be discussed with the adolescent and her family (see Section 4.2 and 4.3 in the Technical Report).

3. *Metformin*

Metformin in addition to lifestyle interventions could be considered in adolescents with a clear PCOS diagnosis or with symptoms of PCOS before a diagnosis is made. This was a conditional low GRADE quality evidence-based recommendation based on a meta-analysis evaluating the effect of metformin versus placebo in women with PCOS. This meta-analysis included 20 RCTs (one RCT in adolescents [95]) and highlighted the beneficial effects of metformin on BMI, waist-to-hip ratio and triglycerides. Metformin doses used in the meta-analysis trials were 1500–1700 mg per day. Side effects were only reported in five RCTs and included mild to moderate gastrointestinal side effects that were self-limiting (nausea, vomiting, diarrhoea, abdominal pain and non-specified gastrointestinal disturbances). There were no reports on vitamin B12 levels in the trials included in the meta-analysis. Additionally, there were three RCTs (one RCT in adolescents [95]) evaluating the effect of metformin versus lifestyle interventions, which showed improvements in testosterone levels with metformin but with very low certainty in effect estimates. There was also a meta-analysis that evaluated metformin versus COCP in adolescents with PCOS, which included four RCTs [95, 99–101] and showed that metformin at a dose of 1700–2000 mg per day was associated with an improvement in BMI when compared to the COCP [98]. There were no studies in adolescents evaluating the effects of different doses of metformin; the only study that evaluated this in 68 adult women with PCOS did not report a difference in weight effects when using 1500 mg or 2550 mg a day of metformin [105] (see Section 4.4 in the Technical Report).

4. *Antiandrogens*

Recommendations suggest the use of the COCP alone with cosmetic therapy for at least 6 months prior to considering antiandrogens. Where COCPs are contraindicated or poorly tolerated, and in the presence of effective forms of contraception, antiandrogens could be considered to treat hirsutism or androgen-related alopecia. The use of effective contraception is essential due to the teratogenic potential of antiandrogens and their impairment of external genital development in male foetuses. This conditional evidence-based recommendation of very low GRADE quality was based mainly on studies in adult women and included the use of flutamide, finasteride or spironolactone alone or in combination with a diet intervention [106–110]. The reported side effects were gastrointestinal, including mild elevation of transaminases. There was only one study including a small number of adolescents with PCOS (*n* = 14) that evaluated finasteride, but it lacked a direct comparison between groups and did not report side effects [111]. There was insufficient evidence to make an evidence-based recommendation in relation to efficacy of specific types of antiandrogens; the trials included small numbers of subjects and some were not RCTs (see Section 4.6 in the Technical Report). This recommendation was also informed by the Endocrine Society guidelines for the management of hirsutism, which recommend the use of cosmetic and COCP therapy as the first-line treatment for hirsutism in women with PCOS [112].

#### Models of care and transition

There was no evidence-based recommendation as the systematic search did not identify any studies evaluating any type of models of care. A practice point for this section recommended that interdisciplinary care needs to be considered for those with PCOS where appropriate and available. Primary care is generally well placed to diagnose, screen and coordinate interdisciplinary care considering that women with PCOS face multiple health problems across the lifespan and see multiple health professionals. Differences in healthcare systems and settings, including rural or urban areas and developing countries, as well as primary care system differences in developed countries were acknowledged. The coordination of interdisciplinary care also allows the screening and management of comorbidities associated with PCOS. Four studies have described models of care in adult women with PCOS in terms of barriers, enablers and patient/healthcare provider satisfaction [113–116]. There were no studies in adolescents with PCOS nor any studies evaluating outcomes following the transition from paediatric to adult care (see Section 2.6 in the Technical Report). Transition to adult care is required not only for adolescents with a clear diagnosis of PCOS but also for adolescents ‘at risk’ of PCOS to ensure their re-evaluation at an appropriate time to review PCOS diagnosis and management.

## Discussion

The Guideline recommendations are the result of extensive international engagement and rigorous, evidence-based processes aligned with international best practice. Whilst the Guideline provided recommendations for adult women and adolescents, this paper summarises the evidence and recommendations relating to adolescents as defined by the World Health Organisation, namely as females aged between 10 and 19 years. It also addresses recommendations relating to gynaecological age, and specifically 8 years post menarche, and the use of pelvic ultrasound in PCOS diagnosis as well as recommendations relating to the re-evaluation of girls that do not meet the diagnostic criteria in adolescence but are considered ‘at increased risk’ of PCOS. There are few evidence-based recommendations relating to adolescents in the Guideline highlighting the limited evidence in this age group. Limitations within studies included poorly defined study populations, challenges in diagnosis around puberty, the different diagnostic criteria used and lack of availability of data for “time post menarche” of subjects included in most of the studies. Research recommendations highlighted these gaps and emphasised the need for greater high-quality research of PCOS during adolescence.

### Diagnosis

The controversies of diagnosing adolescents with PCOS are compounded by a reliance on 95th centiles cut-off points for menstrual cycles or hirsutism, which are not diagnostic of pathology and are derived from highly variable populations and relatively poor-quality data. Additionally, there is a lack of available outcome data on the long-term health impact of PCOS in conditions such as diabetes. Moving forward, there is a need for greater research into how the confluence of diagnostic factors can be separated out to establish a clear clinical syndrome and how these factors predict short- and long-term adverse health outcomes, with studies in this area now underway.

Diagnosis relied on strong consensus recommendations and aimed to avoid delayed, under- or over-diagnosis of PCOS during adolescence. The criteria included a clear definition of irregular menstrual cycles according to time post menarche and clinical hyperandrogenism, including severe acne and hirsutism and/or biochemical hyperandrogenism, after exclusion of other conditions that mimic PCOS. The use of pelvic ultrasound for diagnosis in females less than 8 years post menarche should be avoided due to the overlap with normal pubertal physiology, the lack of specificity of polycystic ovarian morphology for PCOS diagnosis in this age group and the avoidance of transvaginal ultrasound in those not yet sexually active. AMH was not recommended for a diagnosis of PCOS due to a lack of evidence and apparent lack of specificity. Additionally, special considerations need to be given when assessing adolescents for PCOS; importantly, adolescents’ and their family’s preferences and cultural norms should be included in decision-making.

The Guideline recommends recognising adolescents ‘at risk’. The potential for over-diagnosis is minimised by the narrowed diagnostic criteria; however, there is also respectful recognition of the need to avoid delayed and missed diagnosis and a poor diagnosis experience. As such, reassessment is recommended at 3 years post menarche in relation to menstrual irregularity and at 8 years post menarche in relation to the use of pelvic ultrasound to review the polycystic ovarian morphology. This approach recognises a group of adolescents with only one symptom cluster as ‘at risk’ of PCOS, enabling a focus on symptom management whilst avoiding premature labelling and recommending subsequent follow-up.

### Emotional wellbeing

Following a diagnosis of PCOS, healthcare providers should be aware of the likely increased risk of anxiety and depressive symptoms that will benefit from appropriate screening and management. General Guidelines for the assessment and management of these conditions in children and adolescents should be used as there is no specific data referring to adolescent girls with PCOS. Emotional wellbeing is a poorly evaluated area in adolescent girls with PCOS. Clearly, more research is needed to determine the pathophysiology, whether the symptoms start early in adolescence, the optimal treatment and whether these symptoms will influence engagement with management strategies as observed in adult women with PCOS.

### Treatment

Multicomponent lifestyle interventions (diet, exercise, less sedentary behaviour and behavioural strategies) are the first line of treatment, vital in preventing excess weight gain as well as targeting weight management from adolescence. Pharmacologic management for non-fertility indications in PCOS included evidence-based recommendations with data in adolescents. The treatment options for girls considered to be ‘at risk’ of PCOS and those with a clear diagnosis of PCOS include the COCP and metformin. The COCP is recommended for the management of irregular menstrual cycles and/or hyperandrogenism, whereas metformin alone or in combination with the COCP is recommended to manage weight and metabolic comorbidities.

Recognition of a group who are at risk of PCOS but do not meet all diagnostic criteria allows the treatment of adolescent girls with symptoms yet acknowledges the need for future reassessment at or before 8 years post menarche should PCOS features persist. It also highlights the need for appropriate transition from paediatric to adult care.

### Strengths and limitations

The strengths of the Guideline include the extensive international engagement and rigorous processes aligned with international best practice, including systematic evidence review and synthesis (AGREE II, GRADE, National Health and Medical Research Council (NHMRC) and European Society of Human Reproduction and Embryology (ESHRE) criteria), extensive consultation and partnership at all stages with those affected by PCOS and interdisciplinary health professionals and experts, GDG meetings, integration and response to feedback from international consultation across 37 societies, and the latest updates in key evidence raised during public consultation.

A limitation of the Guideline is the lack of a formal analysis of cost effectiveness or economic feasibility; however, the potential impact of cost was considered in the GRADE process. Furthermore, the Guideline followed priorities that were mostly derived from women with PCOS, not adolescents with PCOS (this will be addressed in the next update of the guideline), whilst the group of multidisciplinary healthcare professionals included paediatricians.

Overall, the evidence considered by the Guideline around adolescence was of low to moderate quality and highlighted the need for future high quality research, including longitudinal studies, to identify the early predictors and natural history of PCOS in adolescents and to allow for timely diagnosis and appropriate management during adolescence and in the transition to adult care. Most importantly, the Guideline refined the diagnostic criteria based on the best available evidence and consensus input from all stakeholders and should allow a more accurate diagnosis of adolescent girls with PCOS.

### Translation

The aim of the comprehensive research translation programme that accompanied the release of the Guideline was to improve the timely and accurate diagnosis and management as well as to augment the consistent implementation of evidence-based care worldwide. A recent Cochrane review demonstrates that obtaining patient-provided information improves adherence with guidelines and evidence-based care [117]. Hence, the Guideline translation encompassed a range of co-designed outputs developed with both women and health professionals, including the freely available and rigorously developed AskPCOS app, which is based on the Guideline and is now used across 112 countries. It provides personalised information and a question prompt list for those with PCOS and their families to optimise health professional engagement. The app also provides health literacy-enhancing tools, comprehensive PCOS-related health information, internationally accredited and accessible health professional-accredited courses on the FutureLearn platform, webinars with international expert panels, and freely available e-health information resources [19, 118]. The guideline and resources are available in a range of languages, proven to be important in guideline uptake [119]. This translation initiative contributes to improved patient experience and serves as an exemplar for international collaborative engagement and healthcare impact.

## Conclusion

Extensive international engagement and rigorous processes have refined the diagnostic criteria to avoid the delayed, under- or over-diagnosis of PCOS and have clarified the treatment of PCOS during adolescence. The Guideline is supported by a multifaceted international translation programme with co-designed resources to educate health professionals and empower adolescents and women with PCOS, including an integrated and comprehensive evaluation programme. However, the evidence synthesis and guideline process highlighted the need for further research on PCOS during adolescence.

## Data Availability

All data related to the Guideline, including the full guidelines, the technical report (including comprehensive evidence review and GRADE framework supporting the recommendations), the disclosure of interest process flow chart, the register of disclosure of interests, administrative report, and public consultation comments and developer responses, are freely available [19].
